# The Banana Theft: Assessing the Diagnostic Accuracy of a Culturally Adapted Picture Description Task as a Cognitive Impairment Measure in the Hai District of Kilimanjaro, Tanzania

**DOI:** 10.1192/bjo.2026.11060

**Published:** 2026-06-30

**Authors:** Hannah Humble, Grace Anderson-Saria, Bernard Mbwele, Lachlan Fotheringham, Stella-Maria Paddick

**Affiliations:** 1 Newcastle University, Newcastle upon Tyne, United Kingdom; 2 Anderson Memorial Rehabilitation and Care Organisation (AMRCO), Moshi, Tanzania, United Republic of; 3 University of Dar es Salaam Mbeya College of Health Sciences, Mbeya, Tanzania, United Republic of; 4 Vijiji Tanzania, Mbeya, Tanzania, United Republic of; 5 Cumbria, Northumberland, Tyne and Wear NHS Foundation Trust, Newcastle upon Tyne, United Kingdom; 6 Gateshead Health NHS Foundation Trust, Gateshead, United Kingdom

## Abstract

**Aims:**

Language deficits present early in Alzheimer’s disease, with symptoms ranging from word-finding difficulties to non-coherent speech. These can be assessed in high-income countries using picture description tasks (PDT). However, they are culturally specific, so have a reduced utility in Sub-Saharan Africa (SSA). The dementia incidence in SSA is increasing but there is a substantial diagnostic gap. Therefore, there is a need for accessible and culturally acceptable cognitive measures. Also allowing for detection of impairments at an earlier stage and for use in prevention studies. This study aims to determine the diagnostic accuracy of a culturally adapted PDT in Tanzania.

**Methods:**

This was a cross-sectional validation in socio economically different communities in Kilimanjaro, Tanzania. Eleven PDT scoring methods were adapted from existing literature. Participants were asked: “tell me everything you can see happening in this picture”. Responses were audio-recorded, transcribed and translated. General cognition was assessed using the Identification and Intervention for Dementia in Elderly Africans (IDEA) screen. Participants with a low IDEA score (≤9) underwent a blinded gold-standard clinical dementia assessment alongside 10% of controls (IDEA >9). Visual acuity was also assessed using a Landolt C near vision chart.

**Results:**

A total of 465 individuals ≥60 years were recruited. 378 individuals were included in the analysis due to 37 refusals and 50 methodological/technical exclusions. All PDT scoring methods, apart from implausible details and words per content unit, correlated with the IDEA screen, age, education and frailty. The scoring method of content units (CUs) showed the greatest correlation. Males, those with greater educational attainment, and a previously skilled occupation performed better. The scoring methods with the highest area under the receiver operating characteristic (AUROC) curve for identification of IDEA score ≤9 were CUs 0.740, CUs and additional details 0.742 and total objects 0.764. The prevalence of dementia was 3.79% (n=8/211). The scoring methods with the highest AUROC curve for identification of dementia were global length of text 0.743 and additional details 0.767.

**Conclusion:**

The diagnostic accuracy was acceptable for three scoring methods to identify cognitive impairment as measured by the IDEA and for two methods in identifying clinical dementia diagnosis. A culturally adapted PDT could be used to support assessment of language in those with cognitive impairment, as part of a wider cognitive testing battery. However, dementia prevalence was very low in this community, potentially biasing findings.

